# Editorial Note: Time to initiation of antenatal care and its predictors among pregnant women in Ethiopia: Cox-gamma shared frailty model

**DOI:** 10.1371/journal.pone.0327507

**Published:** 2025-07-03

**Authors:** The PLOS One Editors

This article [1] was identified as one of a series of articles for which the *PLOS One* Editors have concerns about authorship and adherence to the journal’s first and second publication criteria. We regret that the issues were not addressed prior to the article’s publication.
